# Dissecting the Immunological Microenvironment of Glioma Based on IDH Status: Implications for Immunotherapy

**DOI:** 10.3390/cells14131035

**Published:** 2025-07-07

**Authors:** Miyu Kikuchi, Hirokazu Takami, Yukari Kobayashi, Koji Nagaoka, Yosuke Kitagawa, Masashi Nomura, Shunsaku Takayanagi, Shota Tanaka, Nobuhito Saito, Kazuhiro Kakimi

**Affiliations:** 1 Department of Neurosurgery, Graduate School of Medicine, The University of Tokyo, Bunkyo-ku, Tokyo 113-8655, Japan; kikuchimnsu@tokai.ac.jp (M.K.); takamih-nsu@h.u-tokyo.ac.jp (H.T.); yokitagawa-tky@umin.ac.jp (Y.K.); nomura-m@umin.ac.jp (M.N.); nsaito-nsu@m.u-tokyo.ac.jp (N.S.); 2 Department of Neurosurgery, Tokai University School of Medicine, Isehara 259-1193, Kanagawa, Japan; 3 Department of Immunology, Kindai University Faculty of Medicine, Osakasayama 589-8511, Osaka, Japan; yukkoba@med.kindai.ac.jp (Y.K.); knagaoka@med.kindai.ac.jp (K.N.); 4 Department of Neuro-Oncology, International Medical Center, Saitama Medical University, Hidaka 350-1298, Saitama, Japan; stakayanagi@saitama-med.ac.jp; 5 Department of Neurological Surgery, Okayama Graduate School of Medicine, Dentistry, and Pharmaceutical Sciences, Okayama 700-8558, Okayama, Japan; stanaka@okayama-u.ac.jp

**Keywords:** glioma, IDH status, tumor microenvironment, immunotherapy, tumor-infiltrating lymphocytes, immune checkpoint inhibitors, CIBERSORTx, Ecotyper, RNA-Seq

## Abstract

Gliomas, particularly IDH-wildtype ones, are associated with poor prognosis, yet their immunological landscape remains uncertain. We analyzed RNA sequencing data from 55 glioma patients, estimating immune infiltration with CIBERSORTx and immune cell states via Ecotyper. IDH-wildtype gliomas showed significantly higher immune cell infiltration (*p* = 0.002), notably of regulatory T cells (Tregs) and macrophages, and a greater proportion of exhausted T cells compared to IDH-mutant gliomas. Clustering based on immune profiles revealed two groups. Cluster A, enriched for IDH-wildtype cases, exhibited heightened immune infiltration but also marked immunosuppression. Cluster B, which included both IDH-wildtype and mutant cases, showed lower levels of immune infiltration. Tumor-infiltrating lymphocyte (TIL) cultured from IDH-wildtype tumors demonstrated limited expansion following anti-PD-1, a CSF1R inhibitor, or a STAT3 inhibitor treatment, without clear cluster-specific differences. Tumor-reactive TILs were mainly observed in cluster A. These findings highlight that IDH-wildtype gliomas have an immunosuppressive and heterogeneous microenvironment, potentially limiting responses to single-agent immunotherapies. A personalized, multi-targeted approach addressing multiple immunosuppressive mechanisms may be essential to improve immunotherapy outcomes in this aggressive glioma subgroup.

## 1. Introduction

Gliomas are the most common malignant primary brain tumors, accounting for approximately 30% of all primary brain tumors and 80% of malignant cases [1]. Among them, glioblastoma (GBM) is the most aggressive form, characterized by poor prognosis even with standard treatments, including maximal surgical resection, radiotherapy, and temozolomide chemotherapy [2]. The median overall survival for GBM patients remains approximately 18 months [2,3].

Advances in molecular diagnostics have led to significant changes in glioma classification. The 2021 World Health Organization (WHO) classification emphasized the isocitrate dehydrogenase (IDH) mutation status as a critical biomarker [4,5]. IDH-mutant gliomas, often associated with younger age and better prognosis, are now classified separately from IDH-wildtype glioblastomas, which have worse outcomes [6,7,8,9]. IDH mutations alter cellular metabolism by producing 2-hydroxyglutarate, leading to widespread epigenetic changes and contributing to gliomagenesis [10,11]. Despite efforts to improve outcomes, IDH-wildtype GBM remains highly resistant to conventional therapies. Immunotherapy, particularly immune checkpoint inhibitors (ICIs), has revolutionized treatments in other cancers, but has shown limited efficacy on GBM [12,13,14,15,16]. Nevertheless, successful application of ICIs in other solid tumors, including non-small cell lung cancer and melanoma, despite similarly immunosuppressive microenvironments, suggests that overcoming such barriers may also be feasible in glioma [17]. One contributing factor for that is the highly immunosuppressive tumor microenvironment, characterized by the presence of regulatory T cells (Tregs), immunosuppressive macrophages, and exhausted T cells [18,19,20,21].

Emerging evidence suggests that the immune landscape of glioma differs depending on IDH status. IDH-wildtype gliomas exhibit greater immune cell infiltration, but also stronger immunosuppressive features compared to IDH-mutant tumors [22]. However, the detailed characteristics and functional implications of these differences remain uncertain.

In this study, we aimed to comprehensively characterize the immune microenvironment of gliomas based on IDH status, using RNA sequencing (RNA-Seq) and tumor-infiltrating lymphocyte (TIL) cultures. We further investigated whether the immune environment could be modulated through interventions targeting key immunosuppressive pathways.

## 2. Materials and Methods

### 2.1. Patient Selection

We included glioma patients aged ≥18 years who had undergone surgery at the University of Tokyo Hospital between May 2019 and August 2023. Fifty-five cases with preoperative informed consent and sufficient research specimens were analyzed. Clinical data were collected from medical records of the hospital. The study was conducted in accordance with the Declaration of Helsinki and was approved by the Human Genome and Gene Analysis Research Ethics Committee of the University of Tokyo (Approval Nos. G10028 [25 January 2019] and G3545 [12 March 2013]).

### 2.2. Processing of Tumor Specimens

Tumor resection was guided by preoperative magnetic resonance imaging (MRI) and intraoperative microscopy. In cases with indistinct visual tumor margins, resection was performed with the guide of fluorescent area by 5-aminolevulinic acid (5-ALA; SBI Pharmaceuticals, Tokyo, Japan), administered preoperatively [23]. Resected specimens were divided for pathological diagnosis and research purposes, carefully avoiding necrotic or hemorrhagic areas. Fresh tumor specimens allocated for molecular analysis were snap-frozen in liquid nitrogen and stored at –80 °C for subsequent DNA and RNA extraction. Fresh tumor tissues intended for TIL culture were enzymatically dissociated using the Tumor Dissociation Kit, human (Miltenyi Biotec, Bergisch Gladbach, Germany) and the gentleMACS™ Dissociator (Miltenyi Biotec) to prepare fresh tumor digests (FTDs). The FTDs were suspended in CP-1 solution (Kyokuto Pharmaceutical Industrial Co., Ltd., Tokyo, Japan) and cryopreserved in liquid nitrogen tanks until use in subsequent experiments.

### 2.3. DNA and RNA Extraction

Fresh tumor fragments (3–5 mm) were rapidly frozen and stored at −80 °C. DNA and RNA were extracted using the AllPrep RNA/miRNA Universal Kit^®^ (Qiagen, Hilden, Germany). DNA concentration was measured with the Qubit Assay Kit (Thermo Fisher Scientific, Waltham, MA, USA), and RNA quality was assessed by the Agilent 2200 TapeStation (Agilent Technologies, Santa Clara, CA, USA).

### 2.4. Molecular Genetic Diagnosis

Tumor DNA was amplified by PCR using KOD FX Neo (Toyobo, Osaka, Japan) for IDH1 and AmpliTaq GOLD™ DNA Polymerase (Applied Biosystems, Waltham, MA, USA) for IDH2. Mutations were detected by Sanger sequencing.

### 2.5. RNA-Seq

RNA samples were processed at Veritas Genetics (Danvers, MA, USA) with the NEBNext Ultra II RNA Library Prep Kit for Illumina (New England Biolabs, Ipswich, MA, USA) and sequenced on a NovaSeq 6000 system (Illumina, San Diego, CA, USA). Reads were mapped to the GRCh38/hg38 genome using STAR (v2.7.8a) [24], and gene counts were generated using featureCounts (v1.6.4) [25,26]. Expression levels were calculated as FPKM using R v4.1.3 (The R Project for Statistical Computing, https://www.r-project.org, accessed on 10 March 2022).

### 2.6. Analysis of the Tumor Microenvironment

Immune cell composition was estimated using CIBERSORTx in absolute mode with the LM22 signature matrix (https://cibersortx.stanford.edu, accessed on 20 April 2024) [27,28]. Subsequently, immune cell states were characterized by Ecotyper analysis (https://ecotyper.stanford.edu/carcinoma/, accessed on 11 November 2024) [29]. Biological processes and major signaling pathways were assessed by single-sample gene set enrichment analysis (ssGSEA) using Hallmark gene sets from MSigDB v.2024.1 via GenePattern v3.9.11 (https://www.genepattern.org, accessed on 1 August 2024).

### 2.7. TIL Culture

**Figure 1:**
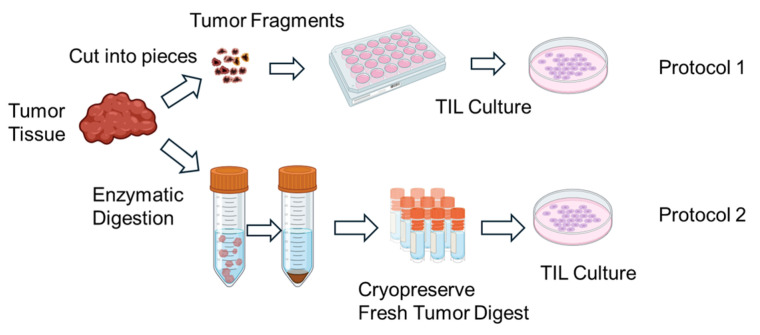
Schematic illustration of the in vitro expansion process of tumor-infiltrating lymphocytes (TILs). Tumor tissues resected from patients are mechanically and/or enzymatically dissociated into small fragments or single-cell suspensions. The resulting tumor-derived materials are then cultured in the presence of high-dose interleukin-2 (IL-2) to promote TIL outgrowth. Over a period of 1–2 weeks, lymphocytes proliferate in culture, resulting in an enriched population of TILs suitable for downstream analyses. Expanded TILs are cryopreserved until use in functional assays. Created in BioRender. Kakimi, K. (2025) https://BioRender.com/u7s0ir5.

TILs were expanded using two different protocols (Figure 1). In Protocol 1, tumor fragments were directly seeded into culture plates without enzymatic dissociation. In Protocol 2, FTDs were seeded at a density of 2 × 10^5^ to 2 × 10^6^ cells per well. TIL cultures were maintained in RPMI1640 medium (Nacalai Tesque, Kyoto, Japan) supplemented with CTS™ Immune Cell Serum Replacement 5% (Gibco, Grand Island, NY, USA), 10 mM HEPES buffer (Dojindo, Kumamoto, Japan), 0.1 mMMEM non-essential amino acids (FUJIFILM Wako, Osaka, Japan), 1 mM sodium pyruvate (FUJIFILM Wako, Osaka, Japan), 14.3 mM 2-mercaptoethanol (Invitrogen, Carlsbad, CA, USA), penicillin (100 U/mL)/streptomycin (100 µg/mL) (Nacalai Tesque, Kyoto, Japan), 100μM indoleamine 2,3-dioxygenase inhibitor (IDOi) called 1-methyl-L-tryptophan (Sigma-Aldrich, MO, USA), and recombinant human interleukin-2 6000 U/mL (IL-2; PeproTech, Cranbury, NJ, USA).

Where indicated, additional reagents were added to the culture medium to modulate the immune environment, including an anti-PD-1 antibody (nivolumab, 10 μg/mL; Ono Pharmaceutical, Osaka, Japan), a colony-stimulating factor 1 receptor (CSF1R) inhibitor (AZD7507, 100 nM; AstraZeneca, Cambridge, UK), a STAT3 inhibitor (AZD9150, 1 μM; AstraZeneca, Cambridge, UK). These agents were added on day 0 of the culture and maintained throughout the expansion period (typically 14 days), with medium changes every 2–3 days. Nivolumab was used to block the PD-1/PD-L1 axis and potentially rescue exhausted T cells. AZD7507 was employed to inhibit CSF1R signaling and suppress immunosuppressive macrophages, while AZD9150, a STAT3 antisense oligonucleotide, was used to attenuate STAT3-mediated immunosuppressive signaling pathways in the tumor microenvironment. These additions aimed to test whether the modulation of the immunosuppressive milieu could enhance TIL expansion and effector function.

### 2.8. Assessment of Tumor Reactivity

TILs (1.0 × 10^5^ cells/well) were co-cultured with autologous FTD for 20–24 h in 96-well round-bottom plates. After incubation, culture supernatants were collected and centrifuged at 300× *g* for 5 min to remove cellular debris. IFN-γ production was quantified using the IFN gamma Human Uncoated ELISA Kit (Thermo Fisher Scientific, Waltham, MA, USA) following the manufacturer’s protocol.

Briefly, 96-well flat-bottom high-binding ELISA plates (Nunc MaxiSorp) were coated overnight at 4 °C with capture antibody specific for human IFN-γ. Plates were then washed and blocked with blocking buffer (1% BSA in PBS) for 1 h at room temperature. Supernatants and standards were added in duplicate and incubated for 2 h at room temperature. After washing, biotinylated detection antibody was added and incubated for 1 h, followed by streptavidin-HRP and TMB substrate. The reaction was stopped using 1 M H_3_PO_4_, and absorbance was measured at 450 nm using a microplate reader. The concentration of IFN-γ was determined based on a standard curve. TILs were considered tumor-reactive if IFN-γ production increased by more than 100 pg/mL upon co-culture with autologous FTD compared to medium-only control.

### 2.9. Statistical Analysis

Statistical analyses were performed using R (version 4.3.1) with RStudio (version 2022.12.0 + 353) as the integrated development environment. Comparisons were conducted by independent sample *t*-tests or Fisher’s exact tests. Survival analysis used the Kaplan–Meier method. Heatmaps and clustering were created with the ComplexHeatmap package, using Euclidean distance and Ward’s method. A *p*-value < 0.05 was considered statistically significant.

## 3. Results

### 3.1. Patient Characteristics

**Figure 2:**
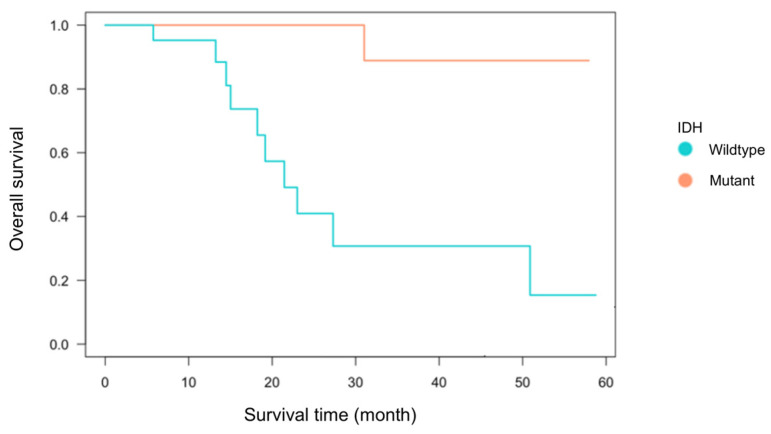
Kaplan–Meier survival curve by IDH status. Kaplan–Meier survival analysis was performed for 38 first-onset cases (22 IDH-wildtype and 16 IDH-mutant cases) using the log-rank test. Although the difference between the two groups was not statistically significant (*p* = 0.07), the IDH-wildtype group tended to exhibit poorer overall survival compared to the IDH-mutant group.

Among the 55 glioma cases analyzed, 33 were IDH-wildtype and 22 were IDH-mutant. The breakdown included 38 primary cases (22 IDH-wildtype and 16 IDH-mutant) and 17 recurrent cases (11 IDH-wildtype and 6 IDH-mutant) (Table 1). There was no significant association between IDH status and recurrence (Fisher’s exact test, *p* = 0.21). The mean age at diagnosis was 59.1 ± 16.0 years for the IDH-wildtype group and 44.0 ± 11.5 years for the IDH-mutant group, consistent with previous reports showing significantly younger onset in IDH-mutant gliomas [1,7,30]. The extent of tumor resection, based on medical record documentation, did not significantly differ between groups: gross total to partial resection was 11:16 in the IDH-wildtype group and 9:11 in the IDH-mutant group. Kaplan–Meier analysis of primary cases demonstrated a trend toward worse overall survival in the IDH-wildtype group (*p* = 0.07, Figure 2), confirming established prognostic differences [7,30]. These findings indicate that the clinical characteristics of this cohort are consistent with prior studies and suitable for immunological analysis.

### 3.2. Immune Cell Infiltration According to IDH Status

**Figure 3:**
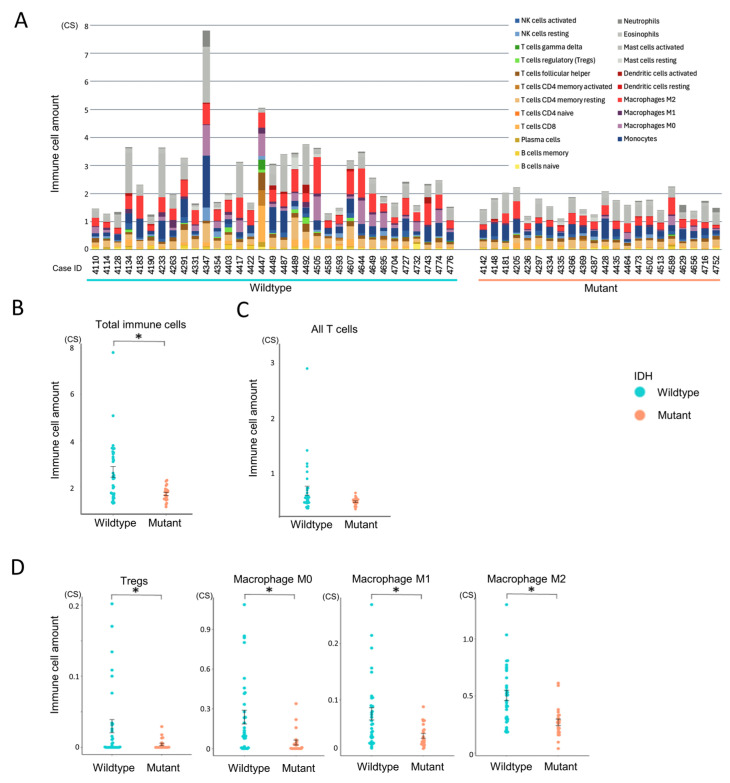
Comparison of immune cell infiltration between IDH-wildtype and mutant groups. Immune cell infiltration patterns were analyzed using CIBERSORTx to assess differences between IDH-wildtype and IDH-mutant gliomas. Statistical comparisons were performed using independent samples *t*-tests. A *p*-value < 0.05 (*) was considered statistically significant. (**A**) Total immune cell infiltration per case estimated using CIBERSORTx absolute mode. (**B**) Comparison of overall immune cell abundance between IDH-wildtype and IDH-mutant groups. (**C**) Comparison of total T cell infiltration between IDH-wildtype and IDH-mutant groups. (**D**) Relative abundance of selected immune cell subsets (regulatory T cells, macrophages M0, M1, and M2) stratified by IDH status.

To evaluate immune infiltration in the tumor microenvironment, we applied CIBERSORTx in absolute mode to RNA-Seq data from all 55 glioma cases (Figure 3A). Unlike the relative mode, which scales cell-type proportions within each sample to sum to one, the absolute mode provides scores in arbitrary units that reflect the estimated abundance of each immune cell type. These scores are proportional to the total immune cell content, allowing for both inter-sample comparisons (e.g., between experimental groups) and approximate intra-sample comparisons of immune cell types. Although the output values do not represent actual cell counts or percentages, they offer a consistent scale for evaluating immune infiltration across and within samples.

Gliomas were predominantly infiltrated by myeloid-derived cells, particularly monocytes, macrophages (especially the M2 phenotype), and mast cells, whereas lymphocyte infiltration was relatively limited (Supplementary Figure S1A). Among lymphocytes, resting memory CD4^+^ T cells were the most abundant, while CD4^+^ naïve T cells and γδ T cells were largely absent. Within this overall context, the IDH-wildtype group exhibited significantly higher total immune cell infiltration compared to the IDH-mutant group (*p* = 0.002, Figure 3A,B). Although T cells tended to be more abundant in the IDH-wildtype group, this difference did not reach statistical significance (*p* = 0.052, Figure 3C). Subtype analysis revealed that Tregs and all macrophage subsets (M0, M1, and M2) were significantly more enriched in the IDH-wildtype group (Figure 3D), whereas other T cell subtypes showed no significant intergroup differences (Supplementary Figure S1B). These findings suggest that IDH-wildtype gliomas harbor a more immunosuppressive tumor microenvironment, characterized by the enrichment of macrophages and Tregs.

### 3.3. Characterization of Immune Cell Functional States

**Figure 4:**
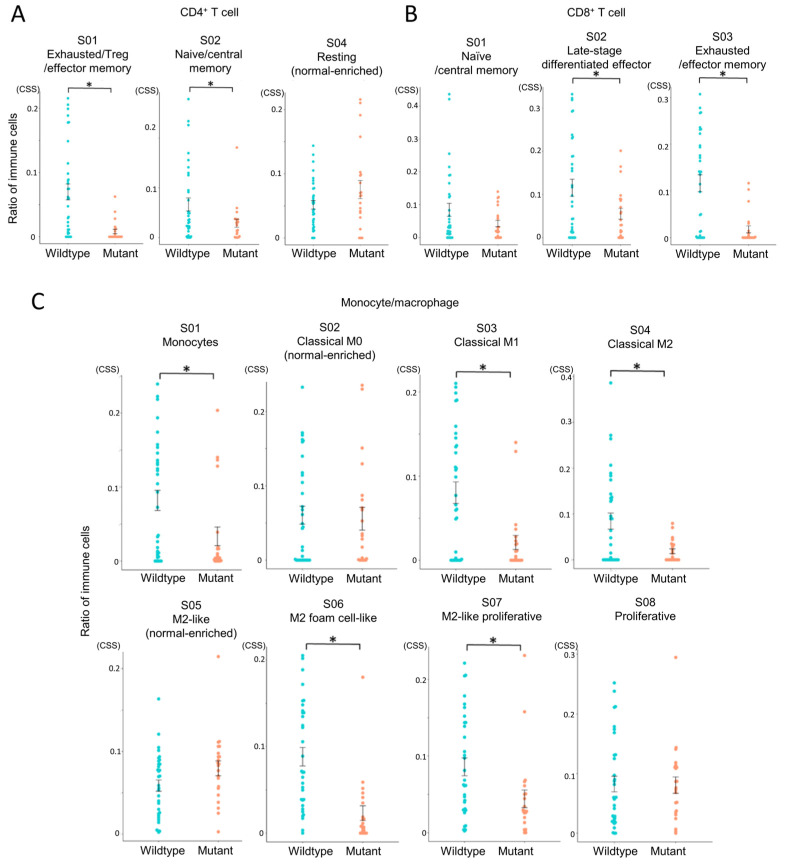
Comparison of immune cell states between IDH-wildtype and IDH-mutant gliomas. Immune cell states in glioma tissues were analyzed using EcoTyper to compare IDH-wildtype and IDH-mutant groups. Cell state scores (CSS) were calculated as unitless, normalized values representing the relative abundance or activity of transcriptionally defined cell states across samples. Higher scores indicate greater representation or activation of a given cell state. Statistical comparisons were performed using independent samples *t*-tests, with *p*-values < 0.05 (*) considered statistically significant. (**A**) Comparison of CD4^+^ T cell states (S01, S02, S04). (**B**) Comparison of CD8^+^ T cell states (S01, S02, S03). (**C**) Comparison of monocyte/macrophage states (S01–S08).

To assess the differentiation and activation states of infiltrating immune cells, we performed Ecotyper analysis (Figure 4). EcoTyper deconvolutes bulk RNA-Seq data into transcriptionally defined cellular states. The resulting cell state scores are unitless, normalized values that reflect the relative abundance or activity of specific cell states across samples. These scores do not represent actual cell counts or proportions, but rather indicate the similarity between a sample’s expression profile and the reference signature of each cell state. Higher scores suggest a stronger presence or activation of the corresponding cell state within a sample. These values are suitable for inter-sample comparisons, clustering, and downstream analyses such as survival or pathway enrichment.

This revealed that exhausted CD4^+^ and CD8^+^ T cell states were more abundant in the IDH-wildtype group compared to the IDH-mutant group (Figure 4A,B), indicating impaired T cell functionality in the IDH-wildtype group. In macrophage subsets, multiple M2-like phenotypes, associated with tumor promotion, were also significantly enriched in the IDH-wildtype group (Figure 4C). Notably, three out of four M2 subsets showed higher abundance in this group, whereas no immune cell subsets were significantly more prevalent in the IDH-mutant group. These results reinforce the immunosuppressive nature of the IDH-wildtype microenvironment.

### 3.4. Immune Cell State-Based Clustering of Gliomas

**Figure 5:**
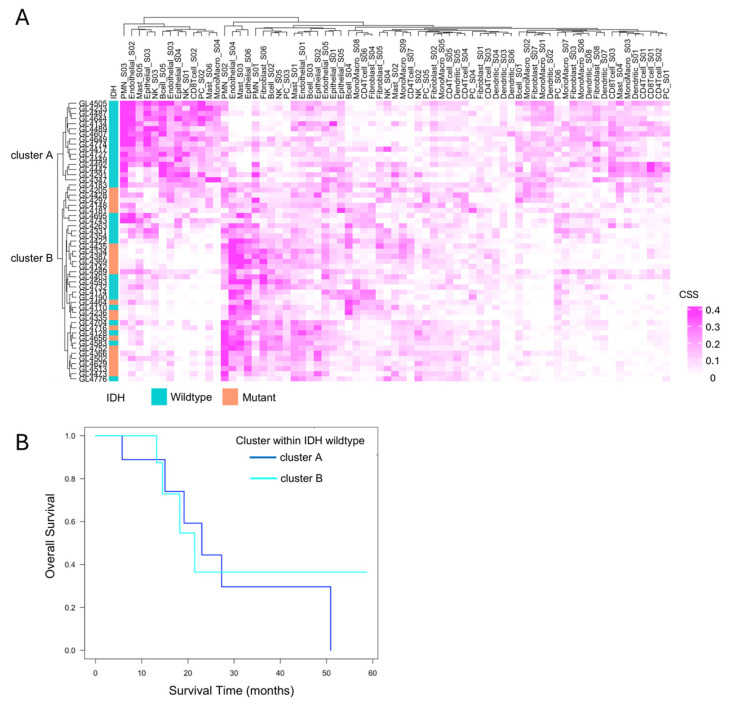
Immune cell state-based clustering and their relevance to the prognosis of IDH-wildtype cases. (**A**) Clustering based on EcoTyper cell states. Hierarchical clustering of all 55 cases based on EcoTyper-derived cell state scores (CSS) classified the samples into two groups: Cluster A and cluster B. All cases in cluster A were IDH-wildtype, whereas cluster B included both IDH-wildtype and IDH-mutant cases. (**B**) Prognosis of primary IDH-wildtype cases by immune cell cluster. The prognosis of 22 primary IDH-wildtype cases was evaluated using the Kaplan–Meier method and the log-rank test, comparing survival between cluster A and cluster B. No significant difference was observed between the two clusters (*p* = 0.4).

To explore immune heterogeneity beyond IDH status, hierarchical clustering was performed based on immune cell state profiles obtained from Ecotyper analysis. This identified two distinct clusters, cluster A and cluster B, characterized by differing immune cell composition and state (Figure 5A). All IDH-mutant cases were classified into cluster B, while IDH-wildtype cases were distributed across both clusters, indicating greater immunological heterogeneity within the IDH-wildtype group. Focusing on the 33 IDH-wildtype gliomas, we assessed the prognostic relevance of this heterogeneity. Kaplan–Meier survival analysis showed no significant difference in overall survival between cluster A and cluster B (*p* = 0.4, Figure 5B).

This suggests that, under current therapeutic regimens, the distinct immune microenvironments—highly inflamed yet suppressed in cluster A versus immunologically “cold” in cluster B—may not influence clinical outcomes. However, these findings do not negate the potential clinical relevance of the immune subtypes. Rather, they highlight that immunological activity alone does not directly translate to survival benefit, especially in the context of T cell exhaustion and immune suppression. Importantly, the immunologically active but suppressed phenotype in cluster A may still represent a population more amenable to immunotherapeutic interventions in the future. Therefore, although cluster A and cluster B exhibit similarly poor prognoses under current therapies, the development of tailored immunotherapeutic strategies targeting the distinct immune characteristics of each cluster may hold promise for improving clinical outcomes in IDH-wildtype gliomas.

### 3.5. Immune Microenvironmental Divergence Within the IDH-Wildtype Group

#### 3.5.1. Quantitative and Compositional Differences in Immune Infiltration

**Figure 6:**
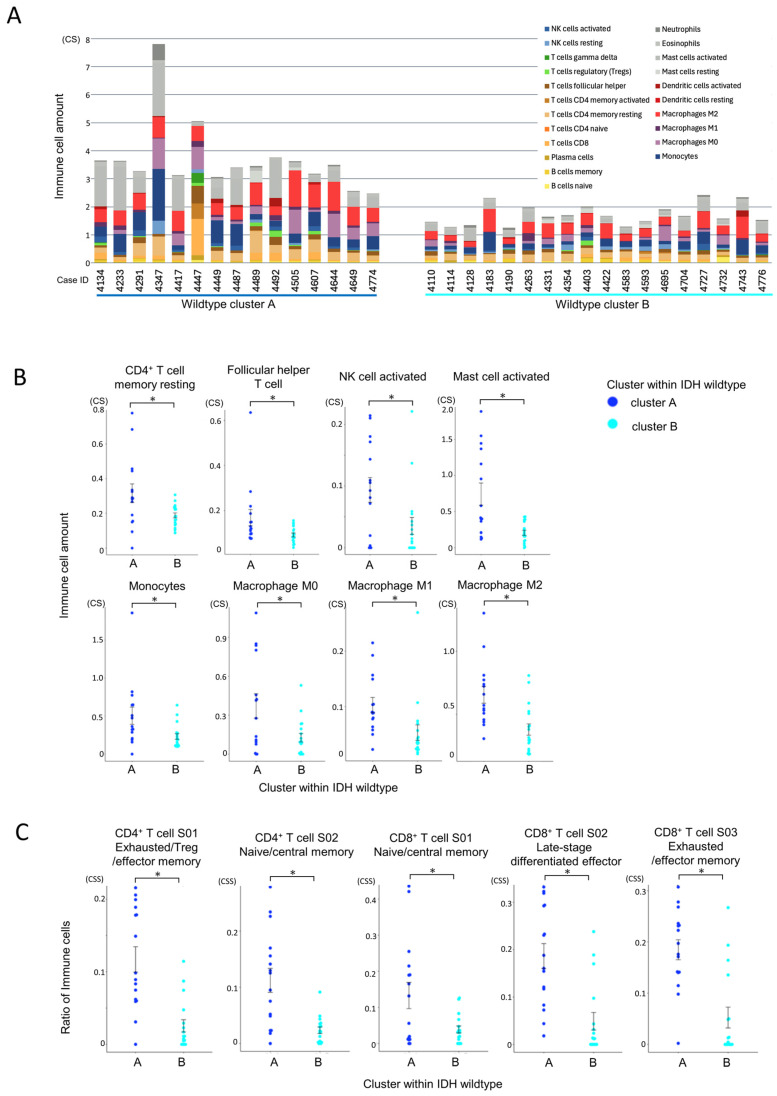
Comparison of immune cell infiltration between IDH-wildtype cluster A and cluster B. Statistical comparisons were performed using independent samples *t*-tests, with *p*-values < 0.05 (*) considered statistically significant. (**A**) Comparison of total immune cell infiltration by CIBERSORTx within the IDH-wildtype group. The total immune cell infiltration per case, estimated using CIBERSORTx, is shown. Cluster A within the wildtype group exhibited a significantly higher total immune cell infiltration than cluster B (*p* < 0.001, independent samples *t*-test). (**B**) Comparison of individual immune cell populations between immune cell clusters in the IDH-wildtype group. The infiltration levels of individual immune cell subsets, estimated using CIBERSORTx, were compared between clusters. CD4^+^ memory T cells (resting) and T follicular helper (Tfh) cells were significantly more abundant in cluster A (*p* = 0.019 and *p* = 0.034, respectively). Monocytes and macrophages (M0, M1, and M2) were also significantly more abundant in cluster A (*p* = 0.020, *p* = 0.013, *p* = 0.017, and *p* = 0.001, respectively). Additionally, NK cell activation and mast cell activation were significantly higher in cluster A (*p* = 0.019 and *p* < 0.001, respectively). (**C**) Differences in T cell states between immune cell clusters within the IDH-wildtype group according to Ecotyper analysis. The cell states of T cells, as defined by Ecotyper, were compared between clusters within the wildtype group. Among CD4^+^ T cells, S01 (exhausted/effector memory/Treg) and S02 (naïve/central memory) states were significantly more prevalent in cluster A (both *p* < 0.001). For CD8^+^ T cells, all cell states analyzed were significantly more prevalent in cluster A (*p* = 0.015, *p* < 0.001, and *p* < 0.001, respectively).

CIBERSORTx analysis revealed that cluster A had significantly greater infiltration of total immune cells and T cells compared to cluster B (Figure 6A). Among immune subpopulations, resting CD4^+^ memory T cells and follicular helper T cells (Tfh) were more abundant in cluster A (Figure 6B). Notably, monocytes and macrophages (M0, M1, and especially M2) were also significantly elevated in cluster A, along with activated NK cells and mast cells. These results suggest a highly infiltrated yet immunosuppressive microenvironment in cluster A.

#### 3.5.2. Functional States of Infiltrating Immune Cells

Ecotyper cell state analysis further demonstrated that exhausted T cell subsets, including both CD4^+^ and CD8^+^ populations, were significantly more prevalent in cluster A (Figure 6C). Despite the higher level of immune infiltration, the presence of dysfunctional T cells and immunosuppressive macrophages suggests impaired anti-tumor immunity in this subgroup.

#### 3.5.3. Pathway Enrichment Analysis Using Hallmark Gene Sets

**Figure 7:**
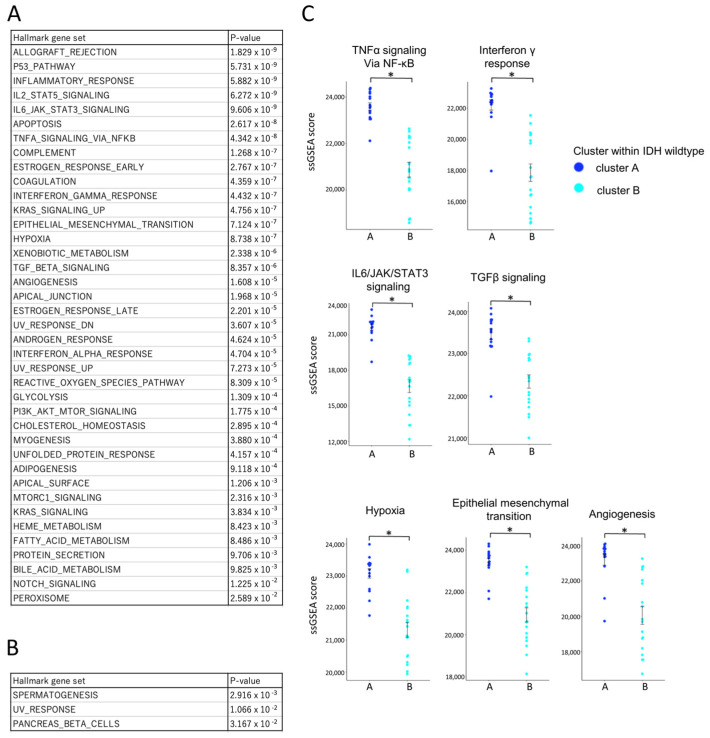
Comparative transcriptomic profiling of IDH-wildtype group clusters A and B using ssGSEA and the Hallmark gene set. Bulk RNA-Seq data from IDH-wildtype cases were analyzed using single-sample gene set enrichment analysis (ssGSEA) with the Hallmark gene sets to compare transcriptomic profiles between cluster A and cluster B within the wildtype group. ssGSEA scores are unitless, scaled relative enrichment scores that indicate the relative activation level of each gene set within a sample. Statistical comparisons were performed using independent samples *t*-tests, with *p*-values < 0.05 (*) considered statistically significant. (**A**) List of gene sets significantly upregulated in cluster A with corresponding *p*-values. Multiple gene sets were significantly upregulated in cluster A compared to cluster B, suggesting a more biologically active tumor microenvironment. (**B**) List of gene sets significantly upregulated in cluster B with corresponding *p*-values. (**C**) Comparison of ssGSEA scores for gene sets associated with key tumor microenvironmental pathways. ssGSEA scores for pathways related to anti-tumor cytokine (TNF-α/NF-κB signaling, IFN-γ response), immunosuppressive cytokine (IL-6/JAK/STAT3 signaling, TGF-β signaling), and immunosuppressive environment (hypoxia, epithelial mesenchymal transition, angiogenesis) were significantly higher in cluster A. An asterisk (*) indicates statistical significance (*p* < 0.05).

To comprehensively assess immune activity, single-sample gene set enrichment analysis (ssGSEA) was performed using Hallmark gene sets (Figure 7A,B). Among the 50 gene sets analyzed, 39 were significantly enriched in cluster A, including those related to TNFα signaling, IFN-γ response, IL6/JAK/STAT3 signaling, TGF-β signaling, hypoxia, epithelial–mesenchymal transition (EMT), and angiogenesis (Figure 7C) [31,32,33,34,35]. Only three gene sets were more active in cluster B. To validate the pathway-level enrichment results, we further compared the expression levels (FPKM values) of representative genes from these pathways between clusters A and B (Supplementary Figure S2). Consistent with the ssGSEA findings, significant differences in gene expression were observed, supporting the robustness of the pathway enrichment analysis. This enrichment indicates that cluster A represents a biologically active, yet immunologically restrained tumor microenvironment.

Collectively, these results define two distinct immune subtypes within the IDH-wildtype group. Cluster A was characterized by a higher overall infiltration of immune and T cells (Figure 6A), with increased proportions of exhausted CD4^+^ and CD8^+^ T cell subsets (Figure 6C) and prominent infiltration of M2-like macrophages (Figure 6B), which are typically associated with immunosuppressive tumor environments. These immunosuppressive features were consistent with the ssGSEA findings, which showed enrichment of pathways associated with immunosuppressive and tumor-promoting processes, including IL-6/STAT3 signaling, TGF-β signaling, hypoxia, EMT, and angiogenesis (Figure 7C).

Together, these findings suggest that although cluster A displays elevated immune activity, the anti-tumor response may be functionally impaired due to the dominance of immunosuppressive components. Based on this, we hypothesized that therapeutic modulation, targeting both macrophage-mediated suppression and T cell exhaustion, could enhance immune function in this subgroup. To test this, we next evaluated the effect of selected immunomodulatory agents during TIL culture.

### 3.6. Evaluation of Tumor Immune Microenvironment in the IDH-Wildtype Group via TIL Culture

#### 3.6.1. TIL Culture Without Drug Addition

To investigate whether immune suppression in gliomas could be modulated ex vivo, TILs were cultured without additional drugs using two protocols. In total, 52 out of 55 cases (31 IDH-wildtype cases and 21 IDH-mutant cases) underwent TIL culture because of limited research specimen.

When comparing the two protocols, Protocol 2 yielded a higher number of cells than Protocol 1 on both IDH-wildtype and mutant cases (Supplementary Figure S3A). We next evaluated the differences in cell yield between IDH-wildtype cluster A and cluster B. Under Protocol 1, there was no significant difference in the number of cells obtained between the two clusters (Supplementary Figure S3B). Similarly, when using Protocol 2, no significant difference was observed between cluster A and cluster B (Supplementary Figure S3C). Overall, these results indicate that while the culture method influences TIL yield, differences between immune subclusters within the IDH-wildtype group were not reflected in the overall proliferation capacity of TILs without drug intervention.

#### 3.6.2. Effect of Anti-PD-1 Antibody on TIL Expansion

**Figure 8:**
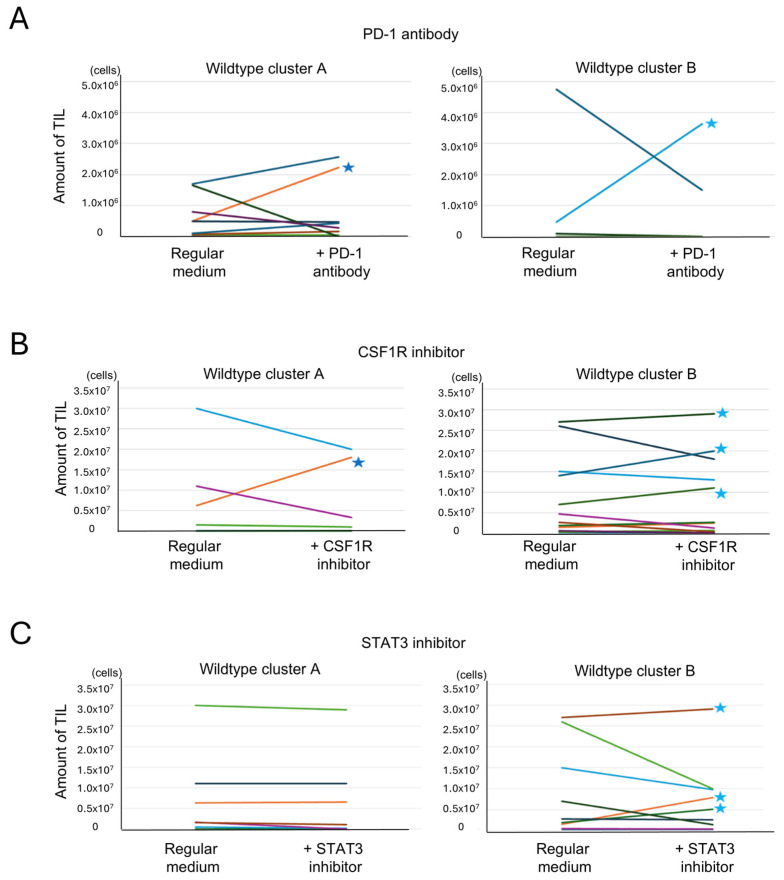
Analysis of the effects of additional drugs on TIL culture. Tumor-infiltrating lymphocyte (TIL) numbers under reagent-treated and untreated conditions are shown. Paired samples from each patient are connected by lines. Each color represents a different patient. (**A**) Effect of an anti-PD-1 antibody addition on TIL culture. TIL culture results with and without the addition of an anti-PD-1 antibody were compared in the same cases. An increase of >10^6^ cells was observed in 1 of 11 cases in cluster A and in 1 of 12 cases in cluster B (indicated by stars (**★**)) (*p* = 0.52, Fisher’s exact test, two-sided). (**B**) Effect of a CSF1R inhibitor addition on TIL culture. TIL culture results with and without the addition of a CSF1R inhibitor were compared in the same cases. An increase of >10^6^ cells was observed in 1 of 6 cases in cluster A and in 3 of 12 cases in cluster B (indicated by stars) (*p* = 0.43, Fisher’s exact test, two-tailed). (**C**) Effect of a STAT3 inhibitor addition on TIL culture. TIL culture results with and without the addition of a STAT3 inhibitor were compared in the same cases. An increase of >10^6^ cells was observed in 0 of 8 cases in cluster A and in 3 of 9 cases in cluster B (indicated by stars) (*p* = 0.12, Fisher’s exact test, two-tailed).

Anti-PD-1 antibody was added to Protocol 1 cultures to assess whether inhibition of PD-1 signaling could enhance TIL proliferation in cases with high T cell exhaustion. Among 23 cases treated with an anti-PD-1 antibody, TIL expansion (>10^6^ cells) was observed in only one case from cluster A and one from cluster B. These cases are indicated with stars in Figure 8A. No significant difference was found between clusters. Interestingly, the cluster A case with TIL expansion exhibited high expression of both PD-1 and PD-L1 (Supplementary Figure S3D), while the responsive cluster B case did not, suggesting that expression levels may influence responsiveness in a subset of cases. These cases are highlighted with red dots in Supplementary Figure S3D.

#### 3.6.3. Effect of CSF1R Inhibitor on TIL Expansion

The CSF1R inhibitor, which targets macrophage differentiation into the M2 phenotype, was added using Protocol 2. Of the 18 cases treated, TIL expansion was observed in one case from cluster A and three from cluster B (Figure 8B). No clear correlation was found between CSF1/CSF1R expression and response to treatment (Supplementary Figure S3E). However, CIBERSORTx analysis showed that cases with TIL expansion had higher levels of monocytes and M1 macrophages, as indicated by the red dot in Supplementary Figure S3F. This suggests that the cellular composition of the tumor microenvironment, rather than ligand expression alone, may influence the efficacy of CSF1R blockade.

#### 3.6.4. Effect of STAT3 Inhibitor on TIL Expansion

To target both macrophages and exhausted T cells, a STAT3 inhibitor was added under Protocol 2. Among 17 treated cases, none in cluster A showed increased TIL proliferation, while three in cluster B did (Figure 8C). Responsive cases in cluster B exhibited high ssGSEA scores for the IL6-JAK-STAT3 pathway, suggesting pathway activity as a potential predictive marker (Supplementary Figure S3G).

#### 3.6.5. Influence of Combined Immune Suppression Pathways on Drug Responsiveness

Although three immune-modulating drugs were tested, no significant differences in TIL expansion were observed between wildtype clusters A and B for any single agent. The initial hypothesis that cluster A, with higher immune suppression, would show greater improvement was not supported (Figure 8A–C). To explore this further, the relationship between immune suppression pathway strength and drug response was analyzed (Table 2). Immune suppression was defined by FPKM scores (for *PDCD1* and *CSF1R*) or ssGSEA scores (for IL6-JAK-STAT3 pathway). Cluster A cases more frequently exhibited strong suppression in two or more pathways, and several such cases showed no increase in TIL proliferation despite drug addition. This suggests that multiple overlapping suppressive mechanisms in cluster A may limit the effectiveness of single-agent therapy. In contrast, cluster B had more cases with weak or no strong suppression, and TIL proliferation was occasionally observed with single-agent treatment. These findings imply that tumors with fewer or weaker suppressive pathways may respond more favorably to immunomodulation.

TIL expansion was achieved in a small subset of cases following drug addition, with variable response across clusters and drug types. In 15 cases of cluster A, only case 4233 recorded the increased TIL amount culturing with drug addition. The limited response in cluster A, despite its high immune activity, is likely due to the presence of multiple converging immune suppression pathways. In cluster B, where suppression was less pronounced, drug responses were more frequently observed; in a total of 5 out of 16 cases (case 4183, 4263, 4704, 4727, 4776), TIL amount increased after drug addition. These results suggest that future strategies should focus on combination therapies targeting multiple immunosuppressive mechanisms to overcome resistance, especially in highly suppressed environments like cluster A. The next step involves evaluating whether cultured TILs exhibit tumor reactivity.

### 3.7. Evaluation of Tumor Reactivity of Cultured TILs

**Figure 9:**
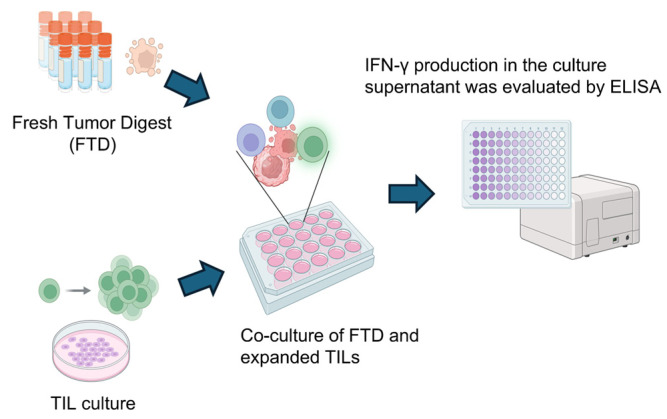
Scheme for analyzing tumor-specific T cell responses in cultured TILs. To detect tumor-specific T cell responses, cultured TILs were co-cultured with FTD from the same case for 20–24 h. The FTD contained tumor cells serving as a source of tumor-associated antigens. As a control, TILs were cultured alone under identical conditions. Following co-culture, IFN-γ levels in the supernatant were measured by ELISA, comparing the TIL-only condition to the TIL + FTD condition. Created in BioRender. Kakimi, K. (2025) https://BioRender.com/sk8bakf.

To assess the tumor reactivity of expanded TILs, IFN-γ production was measured by ELISA following co-culture with autologous FTD containing tumor cells. As illustrated in Figure 9, TILs were stimulated by co-incubation with FTD, and IFN-γ levels in the culture supernatant were used as an indicator of tumor-specific immune response. Although cytokines such as IL-2 and TNF-α are also indicative of immune activation, IFN-γ was selected as the primary marker due to its direct relevance to anti-tumor activity.

The results are summarized in Table 3. Tumor-reactive TILs were defined as those showing increased IFN-γ production upon tumor antigen stimulation and are denoted as “+” (positive) or “−” (negative). In total, 30 cases (4 cases in cluster A of the wildtype group, 11 cases in cluster B of the wildtype group, and 15 cases in the mutant group) with sufficient remaining research specimens proceeded to the evaluation of tumor reactivity. TILs were co-cultured with autologous FTDs, and IFN-γ levels in the culture supernatants were measured by ELISA. The presence of IFN-γ indicated that the TILs were reactive to the tumor. Tumor-reactive TILs were detected only in the IDH-wildtype group. Increased TIL expansion and the presence of tumor reactivity were not always observed in the same cases. Among cases in which tumor-reactive TILs were detected, the distribution between cluster A and cluster B of the wildtype group was 3/4 versus 3/11, respectively (*p* = 0.13, Fisher’s exact test, two-sided). Although the difference was not statistically significant, the proportion was higher in cluster A. This finding is consistent with the greater TIL infiltration observed in cluster A. Nonetheless, the overall prognosis of the IDH-wildtype group remained poorer than that of the IDH-mutant group, implying that the presence of tumor-reactive TILs alone is insufficient to overcome the immunosuppressive tumor environment.

These findings emphasize that, even when tumor-reactive T cells are present, their functional capacity may be suppressed by the glioma microenvironment. The data support the concept that immune suppression in glioma operates through multiple, heterogeneous pathways. Notably, during TIL culture, tumor tissues were enzymatically digested to dissociate cell–cell contacts, thereby reducing suppression mediated by direct interactions with immunosuppressive cells or tumor cells. In addition, the high concentration of IL-2 (6000 U/mL) used in culture likely overcame competition for IL-2 by Tregs [35]. As a result, even T cells that were functionally impaired within the tumor could be expanded in vitro and were able to produce IFN-γ upon re-exposure to autologous tumor digests, indicating preserved tumor reactivity.

## 4. Discussion

This study comprehensively analyzed the tumor immune microenvironment of gliomas based on IDH status using RNA-Seq data and TIL cultures. Consistent with previous findings, IDH-wildtype gliomas exhibited significantly greater immune cell infiltration compared to IDH-mutant gliomas [27,29]. However, this infiltration was characterized by a higher abundance of immunosuppressive components, including Tregs, M2 macrophages, and exhausted T cells. These findings suggest that the poor prognosis associated with IDH-wildtype gliomas may be attributed not simply to the level of immune infiltration, but rather to its suppressive nature.

Using immune cell state data from Ecotyper, we identified two distinct immune subgroups: cluster A, enriched for IDH-wildtype cases with high TIL infiltration and strong immune activation but greater immunosuppression; and cluster B, composed of all IDH-mutant cases and some IDH-wildtype cases, with overall lower immune activity. This subclassification highlights the immunological heterogeneity within IDH-wildtype gliomas.

Compared to conventional methods such as immunohistochemistry and flow cytometry, our approach using bulk RNA-Seq combined with CIBERSORTx and Ecotyper enabled a more detailed and comprehensive profiling of immune cell types and states, including exhausted T cells [22,36,37,38]. While single-cell RNA-Seq provides even greater resolution, it is technically demanding and less feasible in clinical settings [39]. RNA-Seq-based analysis offers a practical and scalable method for immune profiling and may support future clinical application.

In IDH-wildtype cluster A, despite robust immune infiltration, anti-tumor responses appeared limited due to the co-existence of exhausted T cells and M2 macrophages. To address these suppressive factors, we tested an anti-PD-1 antibody, a CSF1R inhibitor, and a STAT3 inhibitor during TIL culture. Although these agents led to increased TIL proliferation in a subset of cases, particularly in cluster B, their overall efficacy was limited in cluster A, where multiple overlapping immunosuppressive pathways were observed. This finding indicates that single-agent immunomodulation may be insufficient in highly suppressed immune environments.

Tumor-reactive TILs were identified exclusively in IDH-wildtype gliomas through co-culture with tumor cell-containing digests and measurement of IFN-γ production by ELISA (Table 3), with a higher frequency observed in cluster A. These findings indicate that tumor-specific T cells are indeed present in the glioma microenvironment. During TIL culture, enzymatic digestion of tumor tissue may have reduced suppression mediated by direct cell–cell contact, and the use of high-dose IL-2 (6000 U/mL) likely helped to overcome IL-2 competition by Tregs [40], allowing even functionally impaired T cells to expand and regain IFN-γ production capacity. However, tumor reactivity did not correlate with clinical prognosis, suggesting that even tumor-reactive T cells are rendered functionally ineffective within the immunosuppressive glioma microenvironment. This underscores the need for strategies that combine immune cell activation with targeted modulation of immune suppression.

While our analyses using CIBERSORTx and Ecotyper provide valuable transcriptomic insights into the immune microenvironment, these methods rely on RNA expression and may not fully reflect protein-level dynamics that determine functional responses. This limitation is particularly relevant to immune checkpoint molecules such as PD-1, PD-L1, CSF1R, and STAT3, whose RNA levels were elevated in cluster A, yet their blockade failed to restore TIL function. These findings suggest a possible discordance between transcriptional signatures and actual protein expression or activity in highly suppressed tumors. Future studies incorporating flow cytometry or immunohistochemistry will be critical to evaluate the expression and localization of these immune modulators at the protein level. Such validation will not only clarify the mechanisms of immune suppression but also help guide rational design of combinatorial immunotherapeutics.

Beyond molecular validation, future experimental approaches, such as co-culture of TILs with 3D tumor organoids or patient-derived glioma spheroids, may provide deeper insights into the tumor–immune interactions within gliomas [41,42]. Additionally, integration of single-cell or spatial transcriptomic technologies could help resolve intratumoral immune heterogeneity and identify localized immunosuppressive niches. These approaches will be instrumental in translating transcriptomic insights into actionable immunotherapeutic strategies.

To acknowledge the limitations of our study more explicitly, we note that the small number of cases available for functional TIL assays limits the generalizability of these findings. Nonetheless, the consistency of the observed patterns across molecular and functional data supports their biological relevance and positions these results as proof-of-concept observations that warrant validation in larger cohorts.

Our findings demonstrate that IDH-wildtype gliomas comprise immunologically distinct subgroups and contain tumor-reactive TILs. However, immune suppression in these tumors is multifaceted and variable across patients. To improve the clinical utility of TIL-based immunotherapy, it will be essential to refine immune profiling methods, develop combination therapeutic strategies, and optimize TIL expansion protocols. In this context, subclassification based on transcriptomic immune features may provide a rational framework for personalized immunotherapeutic approaches. Particularly, patients in cluster A, harboring tumor-reactive but functionally restrained T cells, could benefit from tailored combinatorial strategies that address specific immunosuppressive mechanisms. Future studies integrating transcriptional, functional, and spatial immune profiling will be key to guiding the selection and optimization of individualized immunotherapies in glioma.

## 5. Conclusions

Gliomas, particularly those with IDH-wildtype status, display diverse immune microenvironments characterized by varying levels of immune activation and suppression. While tumor-reactive TILs are present, their function may be compromised by dominant immunosuppressive pathways. This study suggests that therapeutic targeting of exhausted T cells and macrophages—using agents such as anti-PD-1 antibodies, a CSF1R inhibitor, and a STAT3 inhibitor—holds promise, especially in immunologically active subgroups. However, further research is required to overcome resistance mechanisms and translate these findings into effective clinical strategies.

## Figures and Tables

**Table 1:** Patient characteristics.

		Wildtype (N = 33)	Mutant (N = 22)	*p*-Value	Statistical Analysis
Age of onset		59.1	44.0	<0.001	*t*-test
Gender	Male	16	14	0.12	Fisher
	Female	17	8		
Primary or recurrent	Primary	22	16	0.21	Fisher
	Recurrent	11	6		
Overall survival (month)		15.1	29.5	0.07	Log-rank test
Resection	GTR	11	9	0.22	Fisher
	PR	16	11		
	Unknown	6	2		

GTR: Gross total resection, PR: partial resection.

**Table 2:** TIL Culture results and suppression of specific immune regulatory pathways in each case.

ID	IDH	Cluster	Result of TIL Proliferation			Condition of Inhibition				
			Anti-PD-1 Antibody	CSF1R Inhibitor	STAT3 Inhibitor	PD-1 (FPKM)	CSF1R (FPKM)	STAT3 (ssGSEA)	Number of Suppressing Pathways	Category
4134	wild	A	×	×	×	▲	▲	▲	3	‡
4233	wild	A	○	○	×	▲	▲	▲	3	†
4291	wild	A	×	NA	NA	▲	▲	Δ	2	‡
4347	wild	A	×	×	NA	Δ	Δ	▲	1	*
4417	wild	A	×	NA	NA	▲	▲	Δ	2	‡
4447	wild	A	×	NA	×	▲	Δ	Δ	1	‡
4449	wild	A	×	NA	×	Δ	Δ	▲	1	‡
4487	wild	A	×	NA	NA	Δ	▲	▲	2	*
4489	wild	A	×	NA	×	▲	Δ	▲	2	‡
4492	wild	A	×	NA	NA	▲	Δ	▲	2	‡
4505	wild	A	×	NA	NA	▲	▲	▲	3	‡
4644	wild	A	NA	×	×	▲	▲	▲	3	‡
4649	wild	A	NA	×	×	Δ	▲	▲	2	‡
4774	wild	A	NA	×	×	Δ	Δ	Δ	0	*
4110	wild	A	×	NA	NA	Δ	Δ	Δ	0	*
4114	wild	B	×	NA	NA	Δ	Δ	Δ	0	*
4128	wild	B	×	×	×	Δ	Δ	Δ	0	*
4183	wild	B	○	×	○	Δ	▲	Δ	1	§
4190	wild	B	×	NA	NA	Δ	Δ	Δ	0	*
4263	wild	B	×	×	○	Δ	Δ	Δ	0	§
4331	wild	B	×	NA	NA	Δ	Δ	Δ	0	*
4354	wild	B	×	NA	NA	Δ	Δ	Δ	0	*
4403	wild	B	×	×	×	Δ	Δ	Δ	0	*
4422	wild	B	×	×	NA	Δ	Δ	Δ	0	*
4583	wild	B	×	×	×	Δ	Δ	Δ	0	*
4593	wild	B	×	×	×	Δ	Δ	Δ	0	*
4695	wild	B	NA	×	×	Δ	Δ	Δ	0	*
4704	wild	B	NA	○	○	▲	Δ	Δ	1	§
4727	wild	B	NA	○	NA	Δ	▲	Δ	1	†
4732	wild	B	NA	×	NA	Δ	Δ	Δ	0	*
4776	wild	B	NA	○	×	Δ	Δ	Δ	0	§

Symbols: ○: Cases in which the number of cultured TILs increased by >10^6^ cells after the addition of drugs compared to cultures without drug addition. ×: Cases in which no increase in TIL number was observed after drug addition. NA: Cases in which TIL culture was not performed with additional medication. ▲: Cases in which the FPKM value or ssGSEA score ranked among the top 10 out of 31 wildtype cases. Δ: Cases in which the FPKM value or ssGSEA score did not rank among the top 10 out of 31 wildtype cases. *: No pathway suppression was identified, yet TIL expansion did not occur despite drug administration. †: Administration of a drug that inhibits the strongly suppressive pathways resulted in an increased number of cultured TILs. ‡: Administration of a drug that inhibits the strongly suppressive pathways did not increase the number of cultured TILs. §: Administration of a drug that inhibits the weakly suppressive pathways resulted in an increased number of cultured TILs.

**Table 3:** Evaluation of TIL culture results and detection of tumor-reactive TILs.

ID	IDH	Cluster	Result of TIL Proliferation				Result of IFN-γ ELISA				
			No Additional Drug	Anti-PD-1 Antibody	CSF1R Inhibitor	STAT3 Inhibitor	No Additional Drug	Anti-PD-1 Antibody	CSF1R Inhibitor	STAT3 Inhibitor	Tumor Responsive TIL
4233	wild	A	○	○	○	×	-	+	-	-	+
4644	wild	A	○	NA	×	×	+	NA	+	+	+
4649	wild	A	○	NA	×	×	-	NA	-	-	-
4774	wild	A	○	NA	×	×	-	NA	-	+	+
4183	wild	B	○	○	×	○	-	-	-	-	+
4263	wild	B	○	×	×	○	-	NA	-	-	-
4354	wild	B	NA	×	NA	NA	NA	NA	NA	NA	-
4403	wild	B	○	×	×	×	+	NA	+	-	+
4583	wild	B	×	×	×	×	-	NA	-	-	-
4593	wild	B	○	×	×	×	-	NA	-	-	-
4695	wild	B	○	NA	×	×	-	NA	-	-	-
4704	wild	B	○	NA	○	○	-	NA	-	-	-
4727	wild	B	○	NA	○	NA	-	NA	-	NA	-
4732	wild	B	×	NA	×	×	+	NA	NA	NA	+
4776	wild	B	○	NA	○	×	-	NA	-	-	-
4142	mutant	B	NA	×	×	×	NA	NA	NA	NA	-
4148	mutant	B	×	×	×	×	-	-	NA	NA	-
4181	mutant	B	NA	○	×	×	-	-	NA	NA	-
4205	mutant	B	×	×	×	×	-	NA	NA	NA	-
4236	mutant	B	×	×	×	×	-	NA	NA	NA	-
4297	mutant	B	○	×	×	×	-	NA	-	-	-
4366	mutant	B	○	×	×	×	-	NA	-	-	-
4369	mutant	B	×	○	×	×	-	-	-	-	-
4387	mutant	B	×	×	×	○	NA	NA	-	-	-
4435	mutant	B	○	×	×	×	-	NA	-	-	-
4502	mutant	B	○	×	×	×	-	NA	-	-	-
4589	mutant	B	○	×	×	×	-	NA	-	-	-
4656	mutant	B	○	NA	×	×	-	NA	-	-	-
4716	mutant	B	○	NA	×	×	-	NA	-	-	-
4752	mutant	B	×	NA	×	×	-	NA	-	-	-

Symbols: ○: Cases in which an increase of >10^6^ cultured TILs was observed after drug addition compared to without drug addition in the same case. ×: Cases in which no increase in TIL numbers was observed after drug addition. +: Cases in which tumor reactivity was detected by IFN-γ ELISA. -: Cases in which tumor reactivity was not detected by IFN-γ ELISA. (Blank): Cases in which TIL culture or ELISA was not performed. NA: not available.

## Data Availability

The data that support the findings of this study are available from the corresponding author upon reasonable request.

## Supplementary Materials

The following supporting information can be downloaded at: https://www.mdpi.com/article/10.3390/cells14131035/s1, Figure S1: Estimation of Immune Cell Infiltration in Each Case Using CIBERSORTx; Figure S2: Comparison of Representative Gene Expression Levels for Key Pathways Between Cluster A and Cluster B within the IDH-Wildtype Glioma Group; Figure S3: Comparison of TIL Yields, Immune Gene Expression, and Tumor Microenvironment Features Between IDH Subgroups and Clusters.

## Abbreviations

The following abbreviations are used in this manuscript:

CIBERSORTx	Cell type identification by estimating relative subsets of RNA transcripts x
CSF1	Colony stimulating factor 1
CSF1R	Colony stimulating factor 1 receptor
EGFR	Epidermal growth factor receptor
ELISA	Enzyme-linked immuno-sorbent assay
FTD	Fresh tumor digest
GBM	Glioblastoma
ICI	Immune checkpoint inhibitor
IDH	Isocitrate dehydrogenase
IFN-γ	Interferon γ
IL-10	Interleukin-10
IL-2	Interleukin-2
MGMT	O-6-methylguanine-DNA methyltransferase
NK cell	Natural killer cell
OS	Overall survival
PD-1	Programmed death 1
PD-L1	Programmed death ligand 1
PFS	Progression-free survival
RNA-Seq	RNA sequencing
ssGSEA	Single sample gene set enrichment analysis
STAT3	Signal transducers and activator of transcription 3
TERT	Telomerase reverse transcriptase
TGF-β	Transforming growth factor β
TIL	Tumor-infiltrating lymphocyte
Treg	Regulatory T cell
VEGF	Vascular endothelial growth factor
WHO	World Health Organization
