# Supplementary material for: Dissecting the Immunological Microenvironment of Glioma Based on IDH Status: Implications for Immunotherapy
Source: Cells. 2025 Jul 7;14(13):1035. doi: 10.3390/cells14131035 (PMC12249363; doi:10.3390/cells14131035)
Supplement: Supplementary file 1 [file cells-14-01035-s001.zip › cells-3705881-supplementary.pdf]

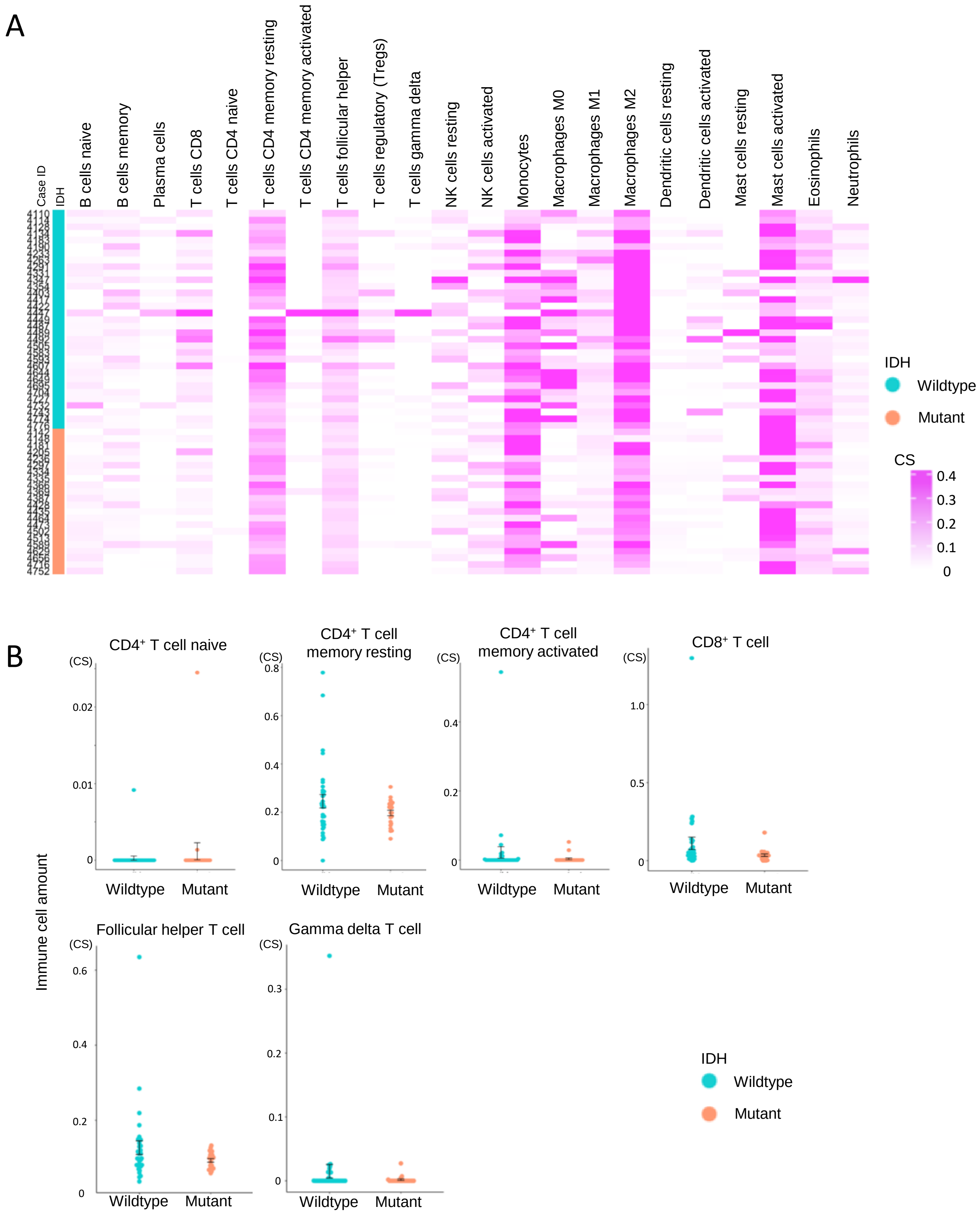

Supplementary Figure S1

Supplementary Figure S1. Estimation of Immune Cell Infiltration in Each Case Using CIBERSORTx.

(A) Immune cell amounts were estimated using CIBERSORTx (absolute mode), yielding unitless scores (indicated in the Figure as CIBERSORTx scores [CS]) that are proportional to absolute cell abundance. These scores enable both inter-sample and intra-sample comparisons of immune infiltration levels. Heatmap analysis of all 55 cases based on CS revealed that M2 macrophages, activated mast cells, monocytes, and resting $CD4^{+}$ memory T cells were the four most abundant infiltrating immune cell types. (B) Comparison of Immune Cell Infiltration Between IDH-wildtype and Mutant Groups. Using CIBERSORTx scores (CS), tumor infiltrating amount of T cell subtypes were compared between IDH-wildtype and Mutant Groups. Except for Tregs (see main Figure 3D), no significant differences were observed in the infiltration levels of other T cell subtypes between the IDH-wildtype and IDH-mutant groups (independent samples t-test).

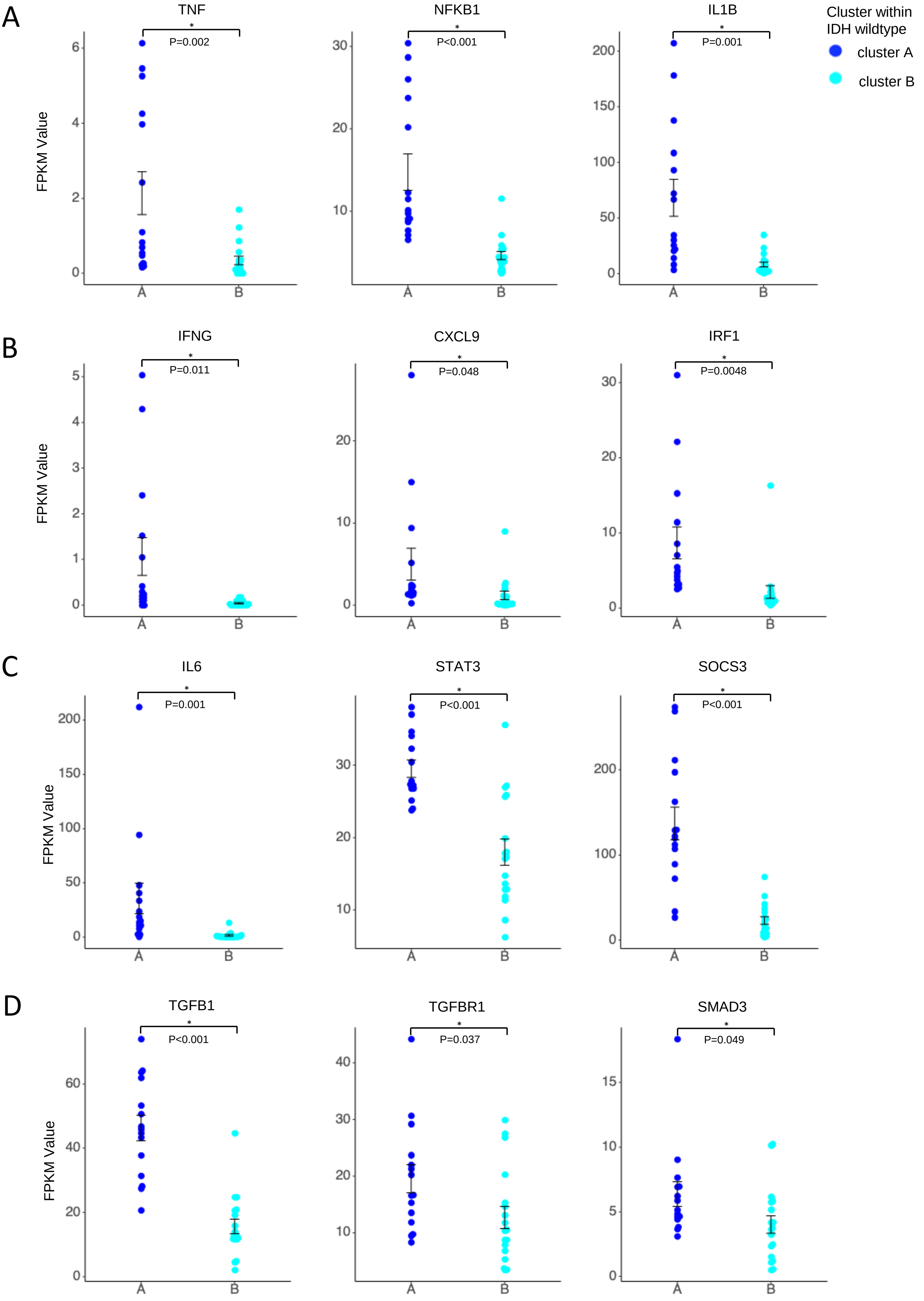

Supplementary Figure S2

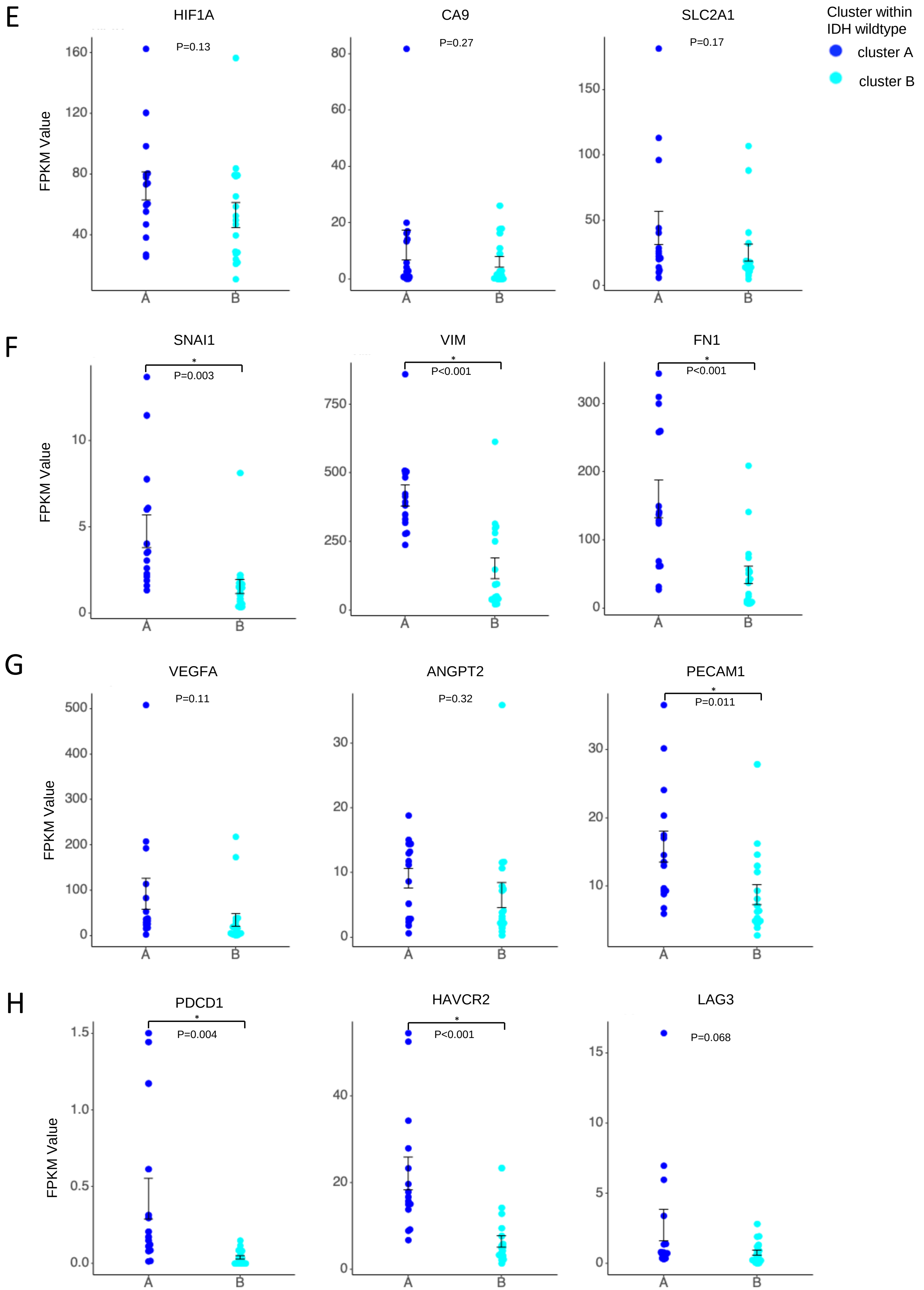

Supplementary Figure S2

Supplementary Figure S2. Comparison of Representative Gene Expression Levels for Key Pathways Between Cluster A and Cluster B within the IDH-Wildtype Glioma Group. The expression levels (FPKM values) of representative genes involved in major immune and stromal pathways were compared between cluster A and cluster B of the IDH-wildtype glioma group. Each panel shows the expression of three key genes related to the indicated pathway:
(A) TNFα signaling: TNF, NFKB1, IL1B
(B) IFN-γ response: IFNG, CXCL9, IRF1
(C) IL6/JAK/STAT3 signaling: IL6, STAT3, SOCS3
(D) TGF-β signaling: TGFB1, TGFBR1, SMAD3
(E) Hypoxia: HIF1A, CA9, SLC2A1
(F) Epithelial–mesenchymal transition (EMT): SNAI1, VIM, FN1
(G) Angiogenesis: VEGFA, ANGPT2,PECAM1
(H) Exhaustion: PDCD1, HAVCR2, LAG3
Independent samples t-tests were used to assess differences in expression between the two clusters. Data are presented as dot plots with mean ± standard deviation. $p < 0.05$ was considered statistically significant.

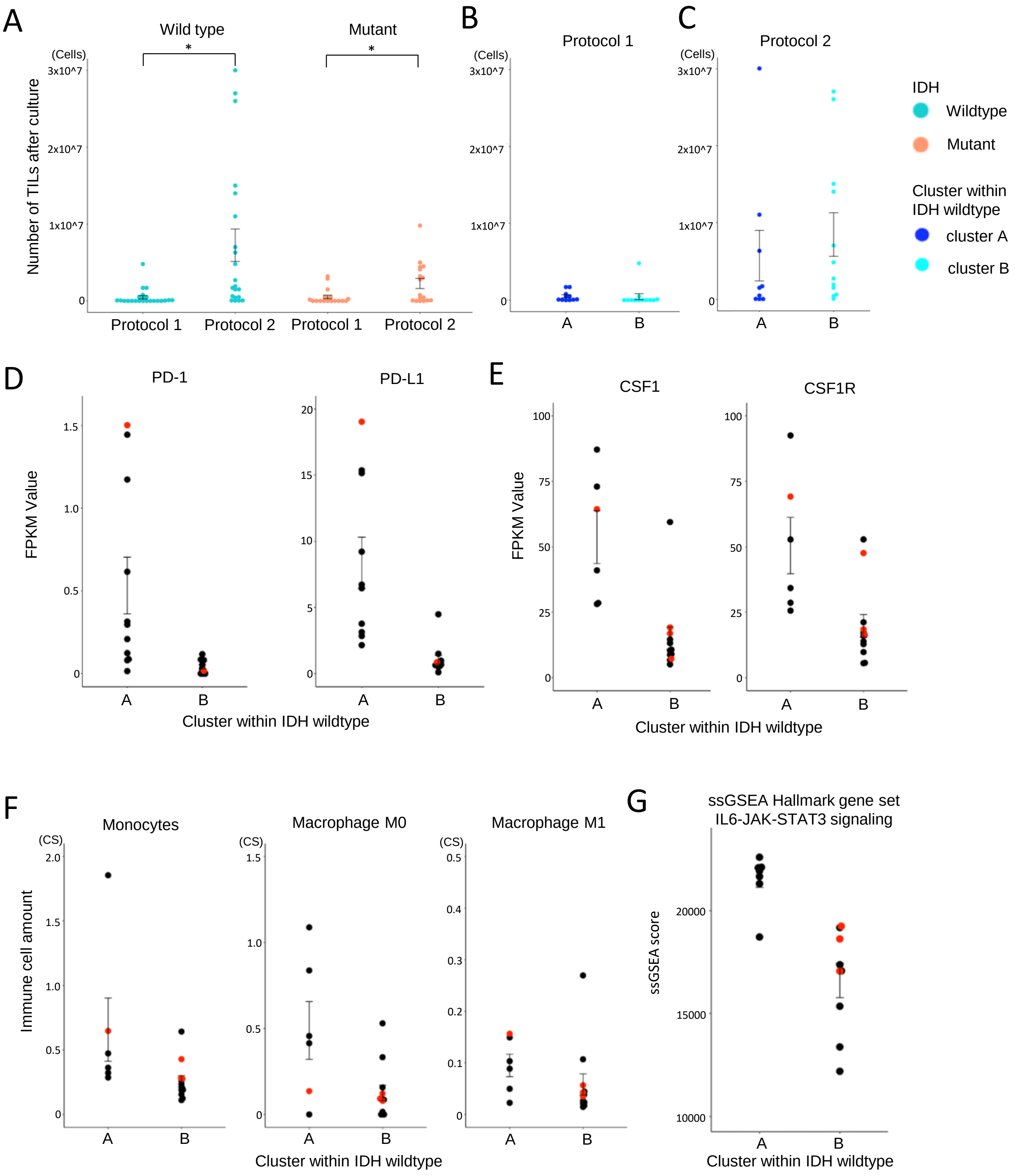

Supplementary Figure S3

Supplementary Figure S3. Comparison of TIL Yields, Immune Gene Expression, and Tumor Microenvironment Features Between IDH Subgroups and Clusters.
(A) Comparison of the number of tumor-infiltrating lymphocytes (TILs) obtained using Protocol 1 and Protocol 2 in IDH-wildtype and IDH-mutant groups. In both IDH-wildtype and IDH-mutant cases, Protocol 2 yielded significantly higher numbers of TILs compared to Protocol 1 (p = 0.002 and p = 0.02, respectively; independent samples t-test).
(B) Comparison of TIL yields between cluster A and cluster B within the IDH-wildtype group using Protocol 1. No significant difference in TIL yields was observed between cluster A and cluster B (independent samples t-test).
(C)Comparison of TIL yields between cluster A and cluster B within the IDH-wildtype group using Protocol 2. No significant difference in TIL yields was observed between cluster A and cluster B (independent samples t-test).
(D)The expression levels (FPKM values) of *PD-1* and *PD-L1* were compared between cluster A and cluster B.
(E) The expression levels (FPKM values) of *CSF1* and *CSF1R* were compared between cluster A and cluster B.
(F) The abundance of monocytes and macrophages, as estimated by CIBERSORTx, was compared between cluster A and cluster B.
(G)The ssGSEA scores for the Hallmark gene set “IL6–JAK–STAT3 signaling” were compared between cluster A and cluster B. Red dots indicate cases with increased TIL yields after drug treatment (panels D–G).
